# Current Situation of the Presence of *Dirofilaria immitis* in Dogs and Humans in Bucaramanga, Colombia

**DOI:** 10.3389/fvets.2020.00488

**Published:** 2020-08-07

**Authors:** María Victoria Esteban-Mendoza, Víctor Arcila-Quiceno, Javier Albarracín-Navas, Isabel Hernández, María Camila Flechas-Alarcón, Rodrigo Morchón

**Affiliations:** ^1^Animal Science Research Group (GRICA), School of Veterinary Medicine and Zootechnics, Master's in Animal Health and Production, Universidad Cooperativa de Colombia, Bucaramanga, Colombia; ^2^Animal and Human Dirofilariosis Group, Department of Faculty, Parasitology Area, Universidad de Salamanca, Salamanca, Spain; ^3^Research Group From Higuera Escalante Clinical Laboratory and Blood Bank, Bucaramanga, Colombia

**Keywords:** *Dirofilaria immitis*, dog, heartworm, human dirofilariosis, prevalence, seroepidemiology, *Wolbachia pipientis*, Colombia

## Abstract

The cardiopulmonary dirofilariosis caused by *Dirofilaria immitis*, is a vector-borne infection, which can be transmitted to humans. The main hosts are both domestic and wild canids. This species mainly occurs in tropical and subtropical climates, and temperature and humidity are the main factors that favor the presence and proliferation of culicid mosquitoes as vectors of the disease. There are few reports of cardiopulmonary dirofilariosis in dogs and humans in Colombia, a region with favorable climatic conditions which favors the presence of mosquitoes that act as vectors of the disease. Therefore, this study aimed to examine its current prevalence in dogs and the risk of human exposure to the disease in Bucaramanga, one of the most populated areas in Colombia located at the center of the country. Furthermore, its demographic and environmental characteristics could be useful as a study model for other similar locations and neighboring countries. Serum samples from 351 dogs and 506 humans from the Bucaramanga Metropolitan area were analyzed. All dog samples were analyzed by Knott's technique and tested with a commercial immunochromatographic to detect the presence of circulating antigens of *D. immitis*. Human samples were analyzed using a non-commercial ELISA test kit to detect IgG against the somatic antigens of adult *D. immitis* and *Wolbachia*. Positive results were further confirmed using western blot analysis. Thirty-eight dogs tested positive with a overall prevalence of 10.82%. Of these dogs, 18 showed *D. immitis* microfilariae, being 5.12% of the total population. The overall seroprevalence in humans was 6.71%; seroprevalence was significantly higher in individuals aged 16–34 years-old and in women than in men. To our knowledge, this study describes seropositivity to *D. immitis* for the first time in a Colombian human population located in the same area as that of dogs infected with *D. immitis*, which represents a potential threat to public health. In humans, age and gender can be considered risk factors for exposure to *D. immitis*.

## Introduction

Cardiopulmonary dirofilariosis, caused by *Dirofilaria immitis*, is a worldwide vector-borne disease in which the definitive hosts are both domestic and wild canines mainly (1). Several species of the genera *Culex* spp., *Aedes* spp., and *Anopheles* spp. are involved in the transmission of this parasite. These species represent a constant risk of infection because they feed on both animal and human hosts (2). Seroprevalence studies in humans have been conducted in regions where infected dogs have been found, which indicated previous contact with the parasite and cases with pulmonary nodules. For this reason, dirofilariosis is considered an emerging public health problem because of its zoonotic potential (3, 4).

Canine cardiopulmonary dirofilariosis is a chronic, progressive, and life-threatening disease. Adult worms are lodged in the pulmonary artery and the right ventricle of a dog's heart. Female mosquitoes ingest the microfilariae, inside which they make two successive molts until third-stage larvae (L3) are inoculated into the definitive host during the next blood meal (2). In humans, immature worms are embolized in the pulmonary microarteries, leading to the formation of benign lung nodules (pulmonary dirofilariosis), of which, most cases are asymptomatic (1, 2). Moreover, *D. immitis* harbors endosymbiotic bacteria of the genus *Wolbachia*, which participate in parasite molting and embryogenesis and play a key role in the immune and inflammatory response to the disease (5, 6).

*Dirofilaria immitis* is primarily distributed in tropical and subtropical climates and depends mainly on environmental factors, including temperature and humidity, in addition to human behavior, such as installation of irrigation systems, taking pets on trips, and new urban developments, that favor the presence and proliferation of its transmission vectors. However, the number of reports in areas with cooler climates has increased, which indicates that the disease is expanding (1, 2, 7, 8).

The South American continent is one of the most biodiverse areas on the planet, with a combination of factors, such as intensification of agricultural practices, landscape modification, poor ecosystem protection, and potentially unstable economies, which lead to the spread of the disease and its vectors (9). The disease has been reported in Argentina, Costa Rica, Venezuela, Peru and Brazil in domestic dogs between others, in where sporadic cases of pulmonary dirofilariosis have been described (1, 10–12). In Colombia, the disease has been reported in dogs from different areas, even in high-altitude areas with cold weather, with mean prevalence values of 0.91–16.12% according to different methodologies (10, 13). However, only one case of human pulmonary dirofilariosis has been described (14), and two seroepidemiological studies were conducted in an area within the Colombian Amazon where infected dogs were also found (15, 16).

The aim of this study was to determine the presence of *D. immitis* in dogs and their possible contact with the human population in the metropolitan area of Bucaramanga, Colombia.

## Materials and Methods

### Sampling Area

**Figure 1:**
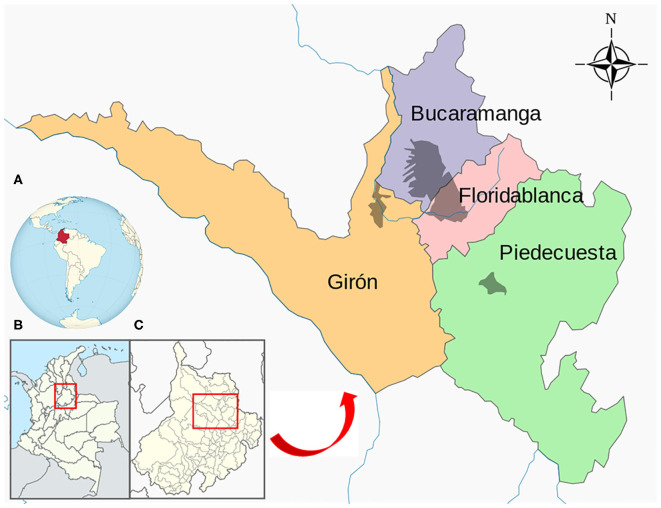
Location of Bucaramanga Metropolitan area, Colombia. **(A)** Colombia; **(B)** Department of Santander (Colombia); **(C)** Bucaramanga Metropolitan area.

Bucaramanga Metropolitan area, which belongs to the capital city of the Department of Santander in Colombia, includes the municipalities of Bucaramanga: Floridablanca, Piedecuesta, and Girón (Figure 1), located near the capital of Colombia, Bogotá. It extends to an area of 1,479 km^2^, and the municipal area occupies 165 km^2^; it is located at 959 m above sea level. The area includes two sectors of different geographical conformations: one formed by a plateau and the other by a valley. Its climate is tropical, with a mean annual temperature of 23.4°C and significant precipitation levels, with an approximate mean annual rainfall of 1,159 mm (17). It has an estimated population of 1.2 million people, and there are 32,000 censused dogs in the city of Bucaramanga alone. In addition, there are numerous uncensored vagrant dogs throughout this area (18).

### Samples Used

This study included samples from 351 dogs and 506 humans collected during February-June 2018. All data collected is shown in Tables 1, 2. The dog and human samples were collected by members of the veterinary staff of different clinics and associations and the Higuera Escalante Laboratory's health care staff, respectively. For the canine population, signed informed consent from the owners was considered as an inclusion criterion. Variables considered for the analysis were gender, age, municipality of residence, socioeconomic status, and whether dogs lived inside or outside of the house. For the human population, being of legal age and signing the informed consent forms were the inclusion criteria. Variables for the analysis were gender, age, municipality of residence, socioeconomic status, living with pets, type of pet (dog or other), and presence of water sources at <200 m. Confidentiality of patient information was always maintained, and all study participants gave their written consent. Socioeconomic stratification was carried out considering six strata: 1, misery; 2, poverty; 3, poverty with some economic resource; 4, middle class; 5, upper middle class; and 6, upper class (19).

**Table 1:** Prevalence in dogs in the Bucaramanga Metropolitan area in terms of gender, age, municipality, socioeconomic status, and their place of permanence.

**Variable**	**No. of samples**	**No. of positive**	**Prevalence (%)**	**95% CI**	**OR**	**No. of mf dogs**	**Prevalence (%)**	**95% CI**	**OR**
**Gender**									
Male	132	14	10.60	0.0474–0.1511	0.97	7	5.30	0.0144–0.925	1.03
Female	219	24	10.95	0.0679–0.1513	1.01	11	5.02	0.0211–0.0794	0.97
**Age (years)**									
<1	31	2	6.45	−0.0271–0.1561	0.97	0	0.00	0.000–0.000	0.00
1–3.9	109	11	10.09	0.0435–0.1584	1.05	5	4.59	0.0060–0.0858	0.94
4–6.9	119	11	9.24	0.0338–0.1357	0.72	7	5.88	0.0106–0.0911	1.04
7–10.9	67	12	17.91	0.0849–0.2733	1.21	5	7.46	−0.0061–0.0956	0.56
11–15	25	2	8.00	−0.0343–0.1943	0.48	1	4.00	0.0056–0.3144	2.31
**Municipality**									
Bucaramanga	144	11	7.60	0.0276–0.1122	0.85	2	1.38	−0.0055–0.0335	0.80
Floridablanca	73	10	13.70	0.0562–0.2178	1.30	2	2.74	−0.0110–0.0657	1.40
Girón	70	9	12.85	0.0482–0.2090	0.45	8	11.42	0.0379–0.1907	0.75
Piedecuesta	64	8	12.50	0.0417–0.2083	1.66	6	9.37	0.0204–0.1671	1.26
**Socioeconomic status**									
Stratum 1	139	16	11.51	0.0614–0.1688	1.02	12	8.63	0.0391–0.1336	1.33
Stratum 2	57	6	10.50	0.0231–0.1874	0.93	1	1.75	−0.0176–0.0527	0.25
Stratum 3	79	10	12.60	0.0516–0.2015	1.25	2	2.53	−0.0101–0.0607	0.37
Stratum 4	75	6	8.00	0.0090–0.1261	0.75	3	4.00	−0.0066–0.0865	1.24
Stratum 5	1	0	0.00	0.000–0.000	0.00	0	0.00	0.000–0.000	0.00
Stratum 6	0	0	0.00	0.000–0.000	0.00	0	0.00	0.000–0.000	0.00
**Place of permanence**									
Indoors	187	17	9.09	0.0493–0.1325	0.82	7	3.74	0.0100–0.0649	0.71
Outdoors	164	21	12.80	0.0718–0.1736	1.21	11	6.70	0.0286–0.1064	1.33
Total	351	38	10.82			18	5.12		

**Table 2:** Seroprevalence of human dirofilariasis in Bucaramanga Metropolitan area, considering seropositivity is defined by the simultaneous positivity of anti-*D. immitis* and anti-*Wolbachia* antibody response.

**Variable**	**No samples**.	**No of seropositive**.	**Seroprevalence (%)**	**95% CI**	**OR**
**Gender**					
Male	159	9	5.66	0.3192–0.8808	0.77
Female	347	25	7.20	0.3939–0.6930	1.29
**Age (years)**					
18–35.9	294	17	5.78	0.2695–0.6905	0.46
36–50.9	118	10	8.47	0.2248–0.7752	1.50
51–65.9	73	7	9.58	0.2823–1.0510	1.05
66–90	21	0	0.00	−5.8531–6.8531	0.94
**Municipality**					
Bucaramanga	189	13	6.87	0.4540–0.9144	1.09
Floridablanca	176	10	5.68	0.2286–0.6805	0.89
Girón	64	2	3.12	−0.4187–1.4187	0.38
Piedecuesta	77	9	11.68	0.3192–0.8808	1.56
**Socioeconomic status**					
Stratum 1	30	6	20.00	0.3016–0.8010	2.14
Stratum 2	144	12	8.33	0.3406–0.8023	2.03
Stratum 3	193	11	5.69	0.2093–0.6055	1.12
Stratum 4	121	5	4.13	0.2630–1.1656	0.80
Stratum 5	16	0	0.00	0.000–0.000	0.00
Stratum 6	2	0	0.00	0.000–0.000	0.00
**Water sources located at** **<200 m from the house**					
Yes	232	15	6.46	0.3734–0.7804	0.93
No	274	19	6.93	0.3692–0.7165	1.06
**Living with pets or not**					
Yes	377	28	7.42	0.4622–0.7552	1.64
No	129	6	4.65	0.1192–0.6808	0.60
Canines	335	26	7.76	0.3760–0.7668	0.58
Other species	171	8	4.67	0.3889–0.6808	1.10
Total	506	34	6.71		

### Knott's Technique

Dogs blood samples were collected in 1 ml K2 EDTA plastic microtubes by applying the modified Knott technique (20) to check whether there were microfilariae in the blood of the animals included in the study.

### Immunological Tests

Dogs and human blood samples were collected in 3 ml vacutainer plastic tubes and centrifuged. The resulting serum was stored at −20°C until further processing. The number of samples analyzed by the different variables and municipality are collected in Tables 1, 2. Dog serum samples were tested for the presence of *D. immitis* antigens using a commercial immunochromatographic test kit (Uranotest Dirofilaria®, Urano Vet SL, Barcelona, Spain; sensitivity: 94.4%, specificity: 100%) according to the manufacturer's instructions. Human samples were analyzed for the presence of *D. immitis* and *Wolbachia* IgG antibodies using a non-commercial ELISA with some modifications (4, 7, 21, 22). 1:100 and 1:40 serum dilutions were used to detect anti-*D. immitis* and anti-*Wolbachia* IgG antibodies, respectively. Goat anti-human IgG (H + L) conjugated to horseradish peroxidase (Sigma-Aldrich, Madrid, Spain) was used at a 1:4,000 dilutions in both cases. Optical densities (OD) were measured at 492 nm. The cut-off point (OD = 0.8 for DiSA and OD = 0.5 for rWSP) was established by calculating the mean value + 3 standard deviations (3SD) of 30 serum samples obtained from dogs and clinically healthy humans (negative controls) who belonged to an area free of *D. immitis*. Human sera were considered positive when both non-commercial ELISAs were positive for the same serum sample. These results were additionally confirmed using western blot analysis performed according to a previously described methodology (23, 24). Both antigenic extracts were subjected to SDS-PAGE in 12% gels under reduced conditions, and proteins were transferred onto nitrocellulose. Human sera were analyzed at a 1:40 dilution and anti-conjugates at a 1:500 dilutions.

### Geospatial Analysis

A geospatial analysis was performed on the population of dogs and humans from the metropolitan area of Bucaramanga through a spatial overlay of positive cases using the SatScan software v.9.6. and the Bernoulli's model with a 95% significance level (999 replications with *P* < 0.05) based on the Monte Carlo statistical significance test. Further, we established clusters, which are areas with a relative risk of infection in dogs and humans, with a maximum size of 50% of the exposed population, based on population census and positive cases. Clusters were imported into the QGIS software version 3.8.0 to be visualized on the study area map.

### Statistical Analysis

Data were analyzed using SPSS 20.0 software for Windows (SPSS Inc./IBM, Chicago, IL, USA). This is a descriptive study applying univariate analysis for the determination of frequencies and a bivariate analysis through Chi-square and odds ratio (OR) estimation, based on which a statistical analysis was performed for the determination of the association between variables. In all cases, the level of significance was established with a *P*-value of < 0.05.

## Results

The overall prevalence of *D. immitis* in dogs was 10.82% (38/351). Of these positive dogs, 18 showed *D. immitis* microfilariae, being 5.12% of the total population. The prevalence of *D. immitis* and of microfilariae broken down by gender, age, municipality (Bucaramanga, Floridablanca, Girón and Piedecuesta), socioeconomic status and place of permanence are provided in Table 1. No statistically significant differences were observed for the variables gender, age, municipality, socioeconomic status, and place of permanence (indoors and outdoors).

**Figure 2:**
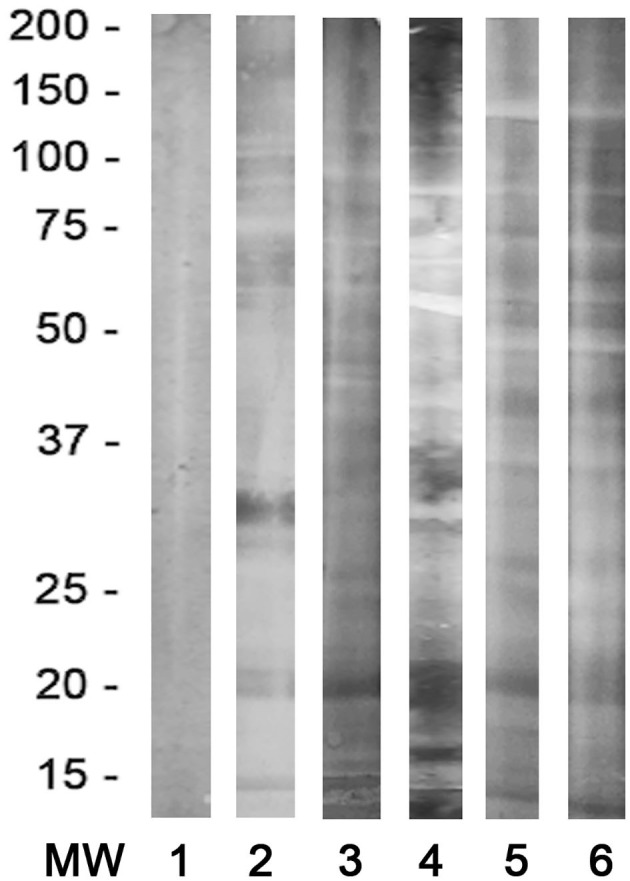
Representative immunoblot analysis of the human sera. Lane 1: negative control; Lanes 2–6: *Dirofilaria immitis*-positive specific bands between 17 and 22 kDa.

The overall seroprevalence in humans was 6.71% (34/506). The seroprevalence broken down by gender, age, municipality (Bucaramanga, Floridablanca, Girón and Piedecuesta), socioeconomic status, water sources located at <200 m from the house and living with pets or not are described in Table 2. All samples positive for western blot analysis are shown in Figure 2.

**Figure 3:**
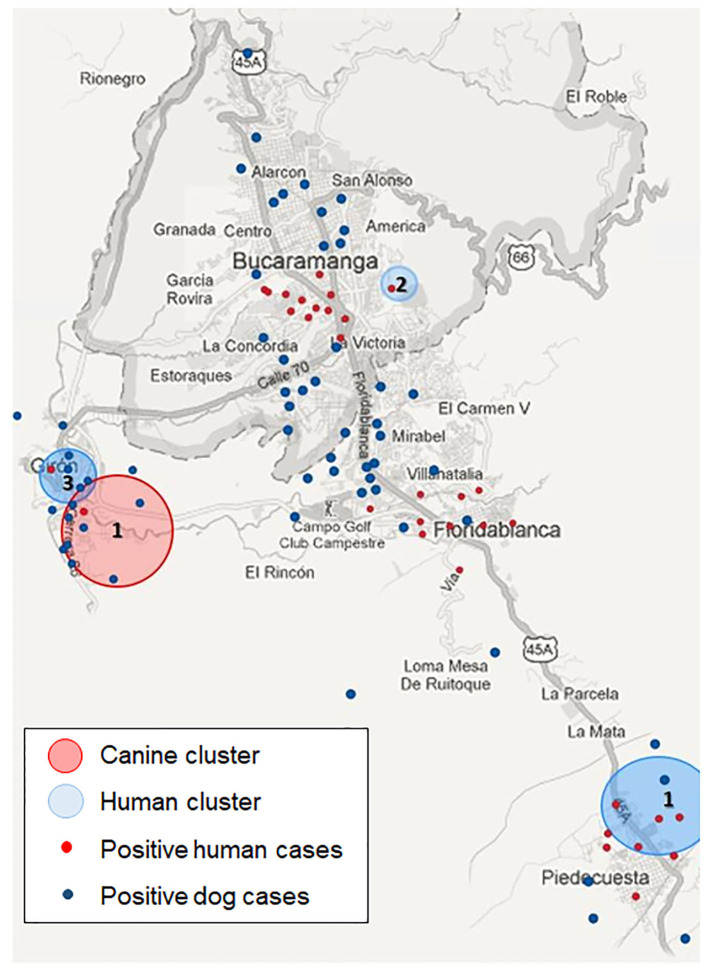
Geospatial exploration of dogs and humans positive for *D. immitis* in the metropolitan area of Bucaramanga imported into Qgis program (version 3.8.0).

In the spatial exploration of dogs positive for *D. immitis* within the study area (Figure 3), 4 significant clusters were detected taking into consideration the magnitude and distribution: one for positive dogs and 3 for seropositive humans (*P* < 0.01).

## Discussion

In this study, we analyzed the presence of *D. immitis* in dogs and humans in the metropolitan area of Bucaramanga, Colombia, analyzing the presence of circulating antigens of *D. immitis* within the canine population, and the response of anti-*D. immitis* and anti-*Wolbachia* IgG antibodies in the human population as a study model for other areas in South America. This first study, to our knowledge of heartworm disease in Bucaramanga, Colombia, revealed an overall prevalence in dogs of 10.82%. Bucaramanga is surrounded by vegetation and is characterized by an average annual temperature of 24°C, high humidity levels, several gullies in its proximity or even in the central areas that accumulate water during the rainy season, and the presence of two rivers (River Oro and River Surata in the areas of Girón and Bucaramanga, respectively). In addition, there are vector species described in the both area, such as *Aedes aegypti*, and other species that could be involved in disease transmission (10, 25). These conditions could promote breeding of these mosquitoes and disease transmission in Bucaramanga. Furthermore, the overall prevalence of microfilaremic dogs was 5.12%, which was heterogeneous. The same situation we can observe in other endemic areas where the disease has been reported (2–4, 7, 8, 10, 26, 27).

Regarding human infections, the overall seroprevalence was 6.71%; to our knowledge, this is the first time that a seroprevalence study for *D. immitis* was carried out in South America. The greatest seroprevalences were detected in Piedecuesta (11.68%) and Bucaramanga (6.87%), where the prevalences in dogs were 12.5 and 7.6%, respectively. We observed slightly lower seroprevalence values of 5.68% in Floridablanca and 3.12% in Girón, where the prevalence observed in dogs was 13.7 and 12.85%, respectively. The occurrence of dirofilariosis in humans depends mainly on the presence of infected dogs and vectors for transmission within a given area. At the same time, other factors, such as an increase in temperature and humidity owing to climate change; emergence of new disease-transmitting species; transportation of infected hosts; modification of the environment owing to human activities; agricultural practices and irrigation areas; deficiency and economic instability; and adverse meteorological events, such as hurricanes or tropical rains in the area, affect the development of the disease (1, 4, 28). Further, it is important to note that although dirofilariosis is a vector-borne disease, not all L3 that come into contact with the host develop into adults, neither in dogs nor in wild hosts (9). Most of the information regarding humans comes from clinical cases and retrospective reviews. In these cases, there are only data from the affected population that showed some type of clinical manifestation, excluding the infected population that does not have symptoms related to the disease or any clinical manifestation, making its study even more difficult. Seroepidemiological studies show the complementary part of the problem, they detect contact with the parasite by analyzing the anti-*Dirofilaria* and anti-*Wolbachia* immune response and are excellent tools to analyze the risk of infection among the human population residing in an endemic area (1, 3, 4, 7, 22, 26, 27).

In Colombia, there has been only one clinical case of a patient from whom an adult worm identified as *Dirofilaria* sp. was extracted from the lung (14), and there are two studies that warn of the existence of human infections caused by *D. immitis* in communities from the Colombian Amazon where infected dogs have been found (15, 16).

Data points related to the geographical location of the samples, and humans with positive serology have been reported in the same location as infected dogs. In addition, spatial clusters in these areas with a relative risk of <1 were detected for humans, suggesting a positive association between the variables studied and a higher frequency of contact with the parasite. These data may suggest a relationship between the presence of *D. immitis*-infected dogs and the seroprevalence detected in humans. This is similar to what occurs in other European countries, such as Spain, Portugal (6.1%), Romania and Moldova (10.9%), and Russia (0.63–4.3%), where the risk of infection among humans has been studied (1, 3, 4, 7, 8, 26, 27).

Regarding the variables evaluated, we observed that age can be a risk factor. In our study, the population with the highest seropositivity was that from the age group of 51–65 years-old. This result is similar to other studies that report that the risk of infection increases with age (3, 4, 7, 8, 26, 27). Furthermore, not only did this study allow us to address the problem from a biological point of view, but also from a socioeconomic point of view in the case of humans. The greatest seroprevalence was observed in stratum 1, where sanitary hygiene conditions are not adequate (20%), followed by stratum 2 (8.3%), stratum 3 (5.7%), and stratum 4 (4.1%). Seropositive individuals were not detected in the last two strata where the sanitary hygiene level is optimal. Socioeconomic status has been associated with mortality and the use of health services, which indicates that a lower income reduces the application of prophylactic and preventive measures to vectors and canines that live with humans (29). Environmental sanitation elements, such as water; sewage, garbage, and waste disposal; sanitary landfills; and garbage treatment, influence the prevalence of parasitosis. These data allow us to associate the lack of sanitary hygiene with the development of dirofilariosis, which may become a socially-determinant public health factor, as in the case of other vector-borne diseases in Colombia, such as malaria, leishmaniosis and Chagas disease (9, 30, 31).

In conclusion, this study describes, for the first time, seropositivity to *D. immitis* and *Wolbachia* Surface Protein in one of the most populated areas of Colombia with a high presence of dogs infected with *D. immitis*. The corresponding authorities should take measures to monitor and control this emerging zoonotic disease to reduce prevalence in canines, while including human pulmonary dirofilariosis in the differential diagnosis of pulmonary nodules. It is necessary to perform further studies in Colombia regarding vectors, reservoirs and humans to clarify the risk of this infection.

## Data Availability Statement

All datasets generated for this study are included in the article/supplementary material.

## Ethics Statement

The sampling process complied with the Helsinki Code of Ethics and Animal Welfare and was approved under resolution no. 040-2019 by the ethics committee of Universidad Cooperativa de Colombia. The participants provided their written informed consent to participate in this study.

## Author Contributions

ME-M, RM, and VA-Q designed the study, wrote the manuscript, participated in the discussion of the results, and corrected the manuscript. JA-N, IH, and MF-A performed the fieldwork and collected the data. All authors read and approved the final manuscript.

## Conflict of Interest

The authors declare that the research was conducted in the absence of any commercial or financial relationships that could be construed as a potential conflict of interest.
